# PCAFA-Net: A Physically Guided Network for Underwater Image Enhancement with Frequency–Spatial Attention

**DOI:** 10.3390/s25061861

**Published:** 2025-03-17

**Authors:** Kai Cheng, Lei Zhao, Xiaojun Xue, Jieyin Liu, Heng Li, Hui Liu

**Affiliations:** 1 Faculty of Information Engineering and Automation, Kunming University of Science and Technology, Kunming 650504, China; 2543666490qq@gmail.com (K.C.); xiaojun35678@kust.edu.cn (X.X.); liheng@kust.edu.cn (H.L.); liuhui621@126.com (H.L.); 2 School of Information Science and Engineering, Southeast University, Nanjing 210096, China; 213223886@seu.edu.cn

**Keywords:** underwater image enhancement, degradation mechanism, frequency and spatial domain

## Abstract

Underwater images frequently experience degradation, including color shifts, blurred details, and reduced contrast, primarily caused by light scattering and the challenging underwater conditions. The conventional methods based on physical models have proven insufficient for effectively addressing diverse underwater conditions, while deep learning approaches are limited by the quantity and diversity of data, making it challenging to perform well in unknown environments. Furthermore, these methods typically fail to fully exploit the spectral differences between clear and degraded images and do not capture critical information in the frequency domain, limiting further improvements in enhancement performance. In order to tackle these challenges, we introduce PCAFA-Net, a physically guided network designed for enhancing underwater images through adaptive adjustment in multiple color spaces and the use of frequency–spatial attention. Our proposed model is made up of three essential modules: the Adaptive Gradient Simulation Module (AGSM), which models the degradation mechanism of underwater images; the Adaptive Color Range Adjustment Module (ACRAM), which adaptively modifies the histogram distributions across RGB, Lab, and HIS color spaces; and the Frequency–Spatial Strip Attention Module (FSSAM), which fully utilizes both frequency and spatial domain information. Extensive experiments were conducted on three datasets, demonstrating that our proposed method outperforms others in both subjective and objective evaluations.

## 1. Introduction

Oceans are vital for sustaining the planet’s ecological equilibrium and supplying a wide range of resources. As natural resources become increasingly scarce, ocean exploration has attracted growing attention. In marine engineering, critical applications such as environmental monitoring, resource exploration, and infrastructure inspection increasingly rely on high-resolution visuals captured by AUVs and ROVs. The observed image can be expressed as shown in Equation (1). Here, $t_{c}(x)$ is the position-dependent transmission coefficient, which determines the attenuation of underwater light and the color shift, and $J_{c}(x)$ and $B_{c}$ are the color components of the original image and the background light, respectively. x represents a pixel location in the image.

(1) $$I_{c}(x)=J_{c}(x)\times t_{c}(x)+B_{c}\times \left(1-t_{c}(x)\right),c\in \{R,G,B\}$$

However, acquiring clear and detail-rich underwater images presents numerous challenges. Underwater images frequently experience degradation, including color shifts, blurred details, and reduced contrast, primarily caused by light scattering and the challenging underwater conditions [1]. It affects clarity, color accuracy, and sharpness, especially at greater depths and in turbid conditions. Hence, UIE methods are essential for improving image clarity in underwater tasks, providing sharper visuals for applications such as monitoring and inspections.

More than a decade ago, physical-model-based methods and non-physical-model-based ones were the mainstream techniques for underwater image enhancement [2]. However, in recent years, with the introduction of convolutional neural networks, researchers in this field have gradually shifted their focus towards deep learning-based UIE methods. The physical-model-based methods leverage prior knowledge to estimate the parameters involved in underwater image formation and subsequently reverse the physical process to improve image quality [3]. However, these assumed parameters do not possess universality in the variable underwater environment. Non-physical-model-based methods primarily adjust the pixel values through image processing techniques to improve aspects like brightness, saturation, and contrast [4]. Despite their effectiveness, these approaches have some drawbacks, such as heavy reliance on specific conditions, potential loss of information, color shifts, and challenges in restoring missing details from the image. With the ongoing advancements in hardware capabilities, deep learning techniques have proven to be very effective in areas such as high-resolution images, segmentation, and object detection, and these innovations have been successfully applied to enhance underwater images [5]. The primary deep learning models used in this domain are CNN and GAN architectures, which utilize their powerful fitting abilities to directly learn the relationship between degraded and real images from training data [6]. Although great progress has been achieved in deep learning-based underwater image enhancement (UIE), many challenges remain unresolved [7]. Most existing approaches focus on improving images in the spatial domain, but the degradation factors affecting underwater images are intricately related within this domain, which can result in uneven enhancement. Inevitably, this may result in situations where the image brightness is increased at the cost of introducing noise or where the clarity of the image is reduced while attempting to improve the color restoration.

In response to these challenges, this paper introduces PCAFA-Net, a physically guided underwater image enhancement network that leverages multi-color space adaptive adjustments and frequency–spatial attention mechanisms. The proposed framework is composed of three key modules: the Adaptive Gradient Simulation Module (AGSM), the Adaptive Color Range Adjustment Module (ACRAM), and the Frequency–Spatial Strip Attention Module (FSSAM). To begin with, we develop the Adaptive Gradient Simulation Module (AGSM) to replicate the physical imaging mechanism of underwater scenes, offering interpretability to the enhancement workflow. Next, the Adaptive Color Range Adjustment Module (ACRAM) is designed to dynamically modify histogram distributions within the RGB, Lab, and HIS color spaces, thereby enhancing image contrast. Additionally, to enable the network to emphasize both the interconnected spatial domain details and crucial frequency domain features, we incorporate the Frequency–Spatial Strip Attention Module (FSSAM), ensuring balanced enhancement across these domains. In conclusion, AGSM provides guidance for deep learning methods that lack sufficient data, while FSSAM compensates for the limitations of traditional methods constrained by specific environments, offering a certain degree of generalization. From the perspective of the frequency domain, FSSAM further refines the factors that lead to color distortion, blurred details, and low contrast in underwater images. AGSM and FSSAM complement each other, and when combined with ACRAM for optimization, they enable PCAFA-Net to enhance degraded underwater images in a balanced manner. Comprehensive test results on both reference and non-reference datasets demonstrate the robust performance and improved optimization capabilities of the proposed network compared to existing learning-based UIE methods. To summarize, the key contributions of this study are outlined as follows:

(1) We propose a novel UIE architecture, PCAFA-Net, which addresses the limitations of traditional UIE methods that are constrained to a single environment and deep learning methods that lack sufficient data, enabling effective restoration of degraded underwater images. Extensive experiments on three datasets demonstrate that PCAFA-Net outperforms other state-of-the-art methods.
(2) Considering the difficulties in physical modeling of traditional UIE methods, we propose the AGSM module, which combines deep learning techniques to automatically learn underwater degradation parameters. We also introduce the ACRAM module that adaptively adjusts histogram distribution, further enhancing image contrast.
(3) Addressing the uneven enhancement issue common in conventional UIE methods, we propose the FSSAM module, which incorporates frequency domain techniques to thoroughly extract degradation factors of underwater images in the frequency domain.

## 2. Related Work

### 2.1. Traditional UIE Methods

#### 2.1.1. Physical-Model-Based Methods

Physical-model-based methods rely on predefined knowledge to infer the unknown parameters of underwater imaging models. These inferred parameters are then integrated into a reverse computational process aimed at enhancing image clarity and restoring visual details. An example can be found in [8], where the authors proposed the Red Channel Method. Berman et al. proposed a method based on haze-lines, introducing a model that accounts for wavelength-dependent light attenuation [9]. Liu et al. [10] introduced an object-guided twin adversarial contrastive learning framework for underwater image enhancement. Peng et al. [11] improved underwater imaging using insights from light absorption. Liang et al. proposed GUDCP, which integrates layered light reflection estimation, robust transmission calculation, and discrete distance-guided color correction to significantly improve underwater image quality [12]. While these physics-based model methods can yield good results in specific situations, their simplified assumptions and the challenge of accurately estimating model parameters make them less effective in complex underwater environments.

#### 2.1.2. Non-Physical-Model-Based Methods

Traditional non-physical-model-based methods enhance the contrast and reduce color distortion in underwater images by modifying the overall pixel values, thus improving the visual appearance. For example, Iqbal et al. [13] employed HSI and RGB color models to correct color balance, improve contrast, and enhance illumination. Ghani et al. [14,15] proposed the DIRS-CLAHS method, integrating contrast adjustments along with color adjustments to enhance underwater image quality. Fu et al. [16] presented a Retinex-based approach for enhancing single underwater images, combining color correction, reflectance, and illumination decomposition. In Garg’s research [17], the paper presented a method of integrating histogram equalization with contrast limitation with percentile techniques. Nevertheless, these approaches may encounter limitations when dealing with complex underwater environments and in obtaining stable, consistent enhancement outcomes.

### 2.2. Learning-Based UIE Methods

Recently, learning-based UIE methods have achieved significant advancements in enhancing underwater images. For example, Wang et al. [18] introduced UIE-Net, a CNN-based model, combining color correction and haze elimination into a single learning framework. Li et al. [19] presented Ucolor, a novel underwater image enhancement network that combines multi-color space encoding and medium transmission-guided decoding. Li et al. [20] introduced a comparative learning framework that explores richer enhancement options through a multi-reference learning strategy and effectively adapts to different image contexts. Islam et al. [21] constructed a generator composed of five encoder–decoder pairs with skip connections. This model achieves fast processing speeds and produces good results in color restoration and sharpening. Zamir et al. [22] presented an effective transformer network aimed at image restoration tasks, such as denoising and deblurring. Peng et al. [23] proposed a novel U-shape Transformer designed for underwater image enhancement, featuring the CMSFFT and SGFMT modules to address channel-specific and spatial inconsistencies in attenuation. Deep learning methods excel in addressing underwater image issues like color distortion, low contrast, and haze. However, most UIE techniques focus on spatial features, neglecting the frequency domain, which holds crucial insights into degradation factors like noise and texture loss. Leveraging this information could significantly improve enhancement performance and visual quality. By analyzing the image in the frequency domain, we can separate the various degradation factors and enhance specific aspects of the image more effectively. This is the main advantage of our method over previous approaches.

## 3. Proposed Method

### 3.1. Overall Pipeline

**Figure 1:**
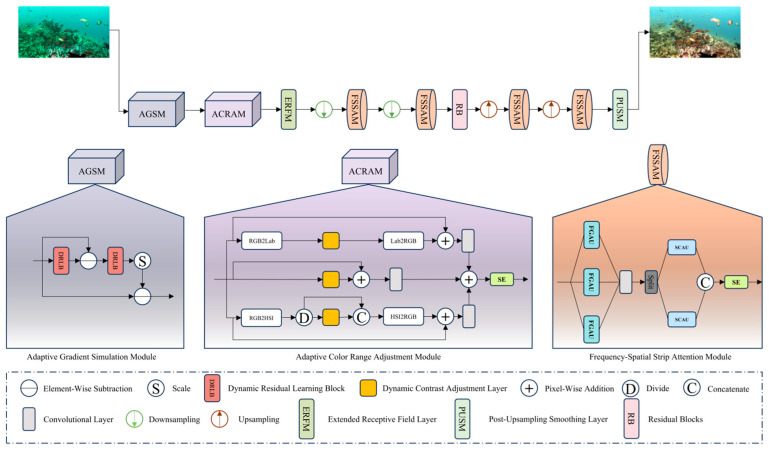
The data-flow and architecture of PCAFA-Net. The network mainly consists of the Adaptive Gradient Simulation Module, the Adaptive Color Range Adjustment Module, and the Frequence-Spatial Strip Attention Module. The Frequency-Spatial Strip Attention Module mainly consists of the Frequency-Guided Attention Unit (FGAU), the Spatial Context Aggregation Unit (SCAU), and Squeeze-and-Excitation Networks (SE). A detailed network module structure is shown in the following subsections.

Our primary goal is to utilize the physical principles of underwater imaging as a guiding framework, integrating both frequency and spatial domains to reveal intricate details and hidden patterns in compromised images. To mitigate color distortion and contrast loss, we also incorporate image processing techniques. Building upon this, we first present the overall workflow of PCAFA-Net, as shown in Figure 1. Following this, we present an in-depth overview of the proposed modules, which include the Adaptive Gradient Simulation Module, the Adaptive Color Range Adjustment Module, and the Frequency–Spatial Strip Attention Module.

Overall Workflow: Initially, the degraded image undergoes processing via the Adaptive Gradient Simulation Module (AGSM), which models the degradation process specific to underwater images. The output from AGSM then passes into the Adaptive Color Range Adjustment Module (ACRAM), where the image undergoes adaptive histogram stretching operations in multiple color spaces. The processed image is then passed through the Extended Receptive Field Layer (ERFL) which includes a reflection padding layer, a 7 × 7 large kernel convolution, normalization, and a ReLU activation function to further extract image features. Subsequently, the image is subjected to two successive downsampling steps and processed by the Frequency–Spatial Strip Attention Module (FSSAM), which combines attention mechanisms from both the frequency and spatial domains to enhance the features extracted earlier. The downsampling process employs a 4 × 4 convolution with a stride of 2 and padding of 1, followed by the application of a LeakyReLU activation function. The image is then processed through residual blocks (RBs), where each block is made up of n sub-blocks. Each sub-block consists of two convolutional layers, preceded by padding layers and followed by normalization layers. A ReLU activation function is applied between the convolutional layers, and dropout layers may also be included to mitigate overfitting and improve the network’s robustness. After this, the image undergoes two additional rounds of repeated upsampling and processing by the Frequency–Spatial Strip Attention Module (FSSAM). Upsampling is carried out using a 4 × 4 transposed convolution layer with a stride of 2, padding of 1, normalization layers, and ReLU activation functions. The image is then processed through the Post-Upsampling Smoothing Layer (PUSL), which includes a reflection padding layer and a 7 × 7 convolution kernel, resulting in the final output image. This sequence of operations produces an enhanced output image.

### 3.2. Adaptive Gradient Simulation Module (AGSM)

The model for underwater image degradation can be formulated as follows:

(2) d=Mc+n

Here, d represents the degraded image observed, c denotes the original clear image that we seek to recover, M is the degradation matrix modeling the underwater degradation process, and n stands for the additive noise term. Traditional model-based methods typically frame UIE as a Bayesian problem, addressing Equation (2) through the lens of maximum a posteriori (MAP) estimation:

(3) $$\hat{c}=\underset{c}{argmax}log\;P(c|d)=\underset{c}{argmax}log\;P(d|c)+log\;P(c)$$

In this context, log P(d∣c) denotes the data fidelity term, while log P(c) represents the regularization term. This optimization problem is then reformulated as an iterative process to find the optimal solution using the Proximal Gradient Descent (PGD) method. The optimization is expressed iteratively as

(4) $${\hat{c}}^{i}=\underset{c}{argmin}\frac{1}{2}{\left\|c-\left({\hat{c}}^{i-1}-\omega \nabla g\left({\hat{c}}^{i-1}\right)\right)\right\|}_{2}^{2}+\lambda J(c)$$

In this expression, the first term corresponds to the gradient descent update, while the second term represents the proximal mapping step, introducing regularization constraints aligned with the updated estimate. Thus, the problem can be broken down into two smaller subproblems:

(5) $$t^{i}={\hat{c}}^{i-1}-\omega M^{⊤}\left(M{\hat{c}}^{i-1}-d\right)\;{\hat{c}}^{i}={prox}_{\lambda ,J}\left(t^{i}\right)$$

Since traditional methods for solving the degradation matrix M not only require a large number of parameters to be manually assumed in advance but also result in biased outcomes, the physical models may favor a specific underwater scenario. To effectively address this challenge, the Adaptive Gradient Simulation Module (AGSM) introduces a gradient estimation-based strategy for adaptively simulating the multiple unknown parameters involved in the degradation process. Additionally, with the help of extensive training data, the AGSM learns the typical characteristics of underwater degradation, allowing it to incorporate this prior knowledge throughout the network.

**Figure 2:**
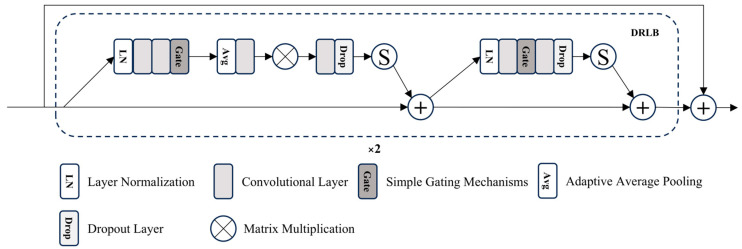
The architecture of the Dynamic Residual Learning Block (DRLB).

The Dynamic Residual Learning Block (DRLB) in AGSM is a crucial component, with one part responsible for simulating the degradation matrix M, and the other part responsible for learning its inverse process $M^{⊤}$. The multi-layer convolution in DRLB provides strong learning capabilities, while its SimGate suppresses irrelevant information. The multiple residual connections ensure that the acquired information is more completed and emphasized. DRLB is shown in Figure 2. The iterative process can be expressed as

(6) $$c^{i}={\hat{c}}^{i-1}-\omega n_{M^{⊤}}^{i}\left(n_{M}^{i}\left({\hat{c}}^{i-1}\right)-d\right)$$

Finally, the physical simulation process of underwater image degradation was performed based on pixel subtraction.

### 3.3. Adaptive Color Range Adjustment Module (ACRAM)

Underwater images are influenced by light attenuation and scattering, which typically result in histogram distributions that are more concentrated. This, in turn, leads to lower contrast and visibility. Traditional histogram stretching often lacks flexibility due to the fixed stretching ratio, which limits its effectiveness. In the previous section, the AGSM based on the physical imaging concept performed preliminary optimization on the degraded image, providing some guidance for the data transmitted to the ACRAM. In this study, we use point-by-point convolution to replace the traditional, manually designed stretching ratios, making histogram stretching trainable to adapt to different underwater scenarios. The expression is as follows:

(7) $${\tilde{I}}_{c}\left(x\right)=\frac{I_{c}(x)-minI_{c}(x)}{maxI_{c}(x)-minI_{c}(x)},c\in \left\{R,G,B\right\}$$

(8) $$I^{r}={Conv}_{1\times 1}\left(\tilde{I}\right)$$

where $\tilde{I}=\left[{\tilde{I}}_{R},{\tilde{I}}_{G},{\tilde{I}}_{B}\right]\in R^{3\times H\times W\;}$ represents the histogram-stretched pixel values in the RGB color space. Global histogram stretching can be viewed as a particular instance of Equation (8).

**Figure 3:**
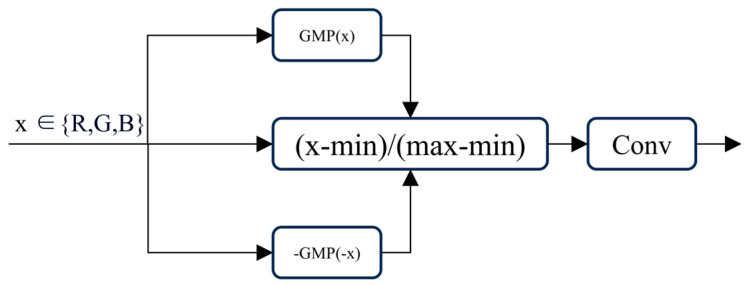
A schematic illustration of the Dynamic Contrast Adjustment Layer (DCAL) in RGB color space.

Figure 3 shows the details of the Dynamic Contrast Adjustment Layer (DCAL) in the RGB space.

In addition to performing histogram stretching in the RGB space, we also transform the image into Lab and HSI color spaces, where histogram stretching is also applied. The Lab color space is developed to approximate human vision, covering a broad spectrum of natural colors and shades. The HSI color space mimics human color perception by representing hue, saturation, and intensity in a natural way. By applying histogram stretching in these three color spaces, the adjustment of degraded images becomes more precise and comprehensive.

Specifically, the ACRAM module adjusts the R, G, B channels, along with the L, a, and b components, and also modifies the S and I channels of the transformed image. It is important to note that the H channel in the HSI color space was not adjusted, as our experiments revealed that modifying the H channel could distort the image’s colors. Finally, the three stretched images are converted back to the RGB color space, each passing through a 3 × 3 convolution layer. The results are subsequently concatenated along the channel axis. The merged output is then passed through an SE attention mechanism [24], which focuses on the combined information from the three color spaces along the channel axis, emphasizing the most significant channel details.

### 3.4. Frequency–Spatial Strip Attention Module (FSSAM)

**Figure 4:**
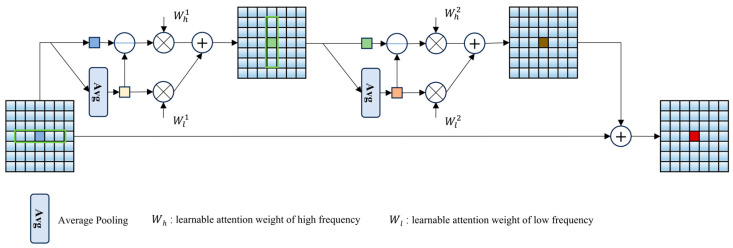
Schematic diagrams of the Frequency-Guided Attention Unit (FGAU). We display only one feature channel for simplicity.

After the processing by the AGSM and ACRAM modules, issues such as color bias, detail blur, and low contrast in the degraded image have been partially resolved, and the model has gained some adaptability to unknown environments. In the FSSAM, we will further refine the degradation factors in the frequency domain, complementing deep learning with traditional methods to balance and enhance the image enhancement effect. The Frequency-Guided Attention Unit (FGAU) in FSSAM aggregates efficient information from the frequency domain. The FGAU is made up of attention mechanisms that operate in both the horizontal and vertical strip directions. Given that the operations in both directions are quite similar, we will focus on explaining the process for the horizontal direction only. Specifically, as shown in Figure 4, given an input tensor $X\in R^{C\times H\times W}$, strip average pooling acts on the input X in the form of a filter to obtain the corresponding low-frequency components. Each strip $S_{c,h,w}\in R^{I\times K}$ is centered around $X_{c,h,w}$, where K denotes the length of the strip. After that, we extract the high-frequency components by subtracting the low-frequency components from the input. Subsequently, learnable attention weights at the channel level are applied to both the low- and high-frequency components, allowing the network to selectively emphasize different frequency information. Ultimately, the result of the horizontal strip attention is derived by combining enhanced low-frequency and high-frequency information.

**Figure 5:**
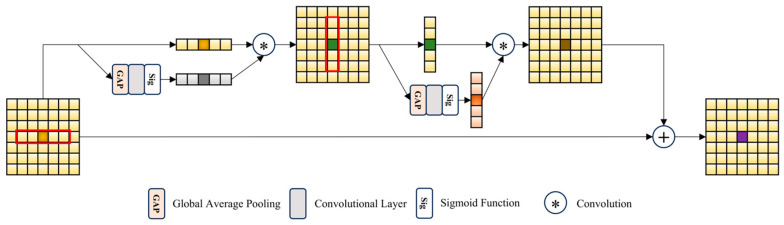
A schematic diagram of the Spatial Context Aggregation Unit (SCAU). We display only one feature channel for simplicity.

The Spatial Context Aggregation Unit (SCAU) module in FSSAM performs efficient information aggregation in the spatial domain. The SCAU also consists of attention operators that work in both the horizontal and vertical strip directions. Given that the operations in both directions are quite similar, we will focus on explaining the details of the horizontal operation. Specifically, as shown in Figure 5, we first apply an extremely lightweight branch to the input $X\in R^{C\times H\times W\;}$ to generate attention weights. This branch is composed of global average pooling (GAP), followed by a 1 × 1 convolution and a Sigmoid activation function. Then, the generated attention weights are multiplied by the corresponding scan area of the strip to obtain the result in the horizontal direction. By combining information from both the horizontal and vertical directions, each pixel implicitly captures the context of the entire K × K region.

Finally, we integrate the FGAU and SCAU at different scales to form our FSSAM module. Specifically, as illustrated in Figure 1, the input features are initially passed through three FGAUs, each utilizing a different strip size. Then, a 1 × 1 convolution is used to further refine the aggregated features from the three frequency branches. The obtained features are subsequently divided into two equal segments along the channel axis. Next, the two segments are fed into separate SCAUs, with K = 7 and K = 11 applied for spatial modulation. Subsequently, the two outputs from the SCAUs are concatenated and undergo additional modulation via an SE attention mechanism to produce the final output of the FSSAM.

### 3.5. Loss Function

The loss function combined the pixel content loss, SSIM loss, and semantic content loss with equal weights (1:1:1) to emphasize pixel accuracy, structural similarity, and semantic relevance, as shown in Equation (9).

(9) $$L=L_{\mathrm{pcont}\;}+L_{\mathrm{ssim}\;}+L_{\mathrm{scont}\;}$$

The pixel content loss directly measures the pixel difference between the restored image $Y_{out}$ and the ground truth image $Y_{ground},$ as shown in Equation (10).

(10) $$L_{\mathrm{pcont}\;}=E\left[{\left\|Y_{out}-Y_{ground}\right\|}_{1}\right]$$

SSIM loss utilizes the commonly used SSIM metric in image restoration to measure the difference between the restored image $Y_{out}$ and the ground truth image $Y_{ground}$ in terms of brightness, contrast, and structural information. The closer the SSIM is to 1, the better the restoration of the degraded image, but this conflicts with the minimization of the objective function. Therefore, we made a slight modification, as shown in Equation (11). And, Equation (12) provides the specific calculation method for SSIM.

(11) $$L_{ssim}=E\left[\frac{1-SSIM\left(Y_{out},Y_{ground}\right)}{2}\right]$$

(12) $$SSIM(x,y)=\frac{\left(2\mu_{x}\mu_{y}+c_{1}\right)\left(2\sigma_{xy}+c_{2}\right)}{\left(\mu_{x}^{2}+\mu_{y}^{2}+c_{1}\right)\left(\sigma_{x}^{2}+\sigma_{y}^{2}+c_{2}\right)}$$

Semantic content loss primarily uses deep convolutional neural networks to extract high-level features to measure the semantic difference between the restored image and the ground truth image. Its mathematical expression is shown in Equation (13), where $k_{i}$ is the i-th weight used for the weighted sum, and $\Omega_{i}\left(Y_{out}\right)$ and $\Omega_{i}\left(Y_{\mathrm{ground}}\right)$ represent the feature maps extracted from the i-th convolutional layer of the pre-trained VGG19_BN network for the restored image and the ground truth image, respectively. MAE (Mean Absolute Error) is the L1 loss between the two feature maps.

(13) $$L_{\mathrm{scont}\;}=\sum_{i=1}^{5}\;k_{i}\cdot MAE\left(\Omega_{i}\left(Y_{out}\right),\Omega_{i}\left(Y_{\mathrm{ground}}\right)\right)$$

## 4. Experimental Discussion

### 4.1. Experimental Settings

#### 4.1.1. Datasets

PCAFA-Net employed several publicly available datasets, such as the Large-Scale Underwater Image Dataset (LSUI), the Underwater Image Enhancement Benchmark (UIEB), and U45. The LSUI dataset is a large-scale collection comprising 4279 real-world underwater images with corresponding reference images [25]. Of these, we randomly selected 3879 pairs for training the model, while the remaining 400 pairs were reserved for testing. Similarly, the UIEB dataset contains a total of 950 underwater images, of which 890 are paired with high-quality reference images. From this set, 800 paired images were utilized for training, with the remaining 90 pairs were allocated for testing [26]. And, Li et al. selected 45 images from real underwater scenes to construct a dataset named U45 [27]. The images were categorized into three subsets according to their degradation levels, which include low contrast and color shifts due to blurring effects: green, blue, and fog subsets. To further verify the model’s generalization capability, we evaluated its performance on the C60 dataset, which comprises underwater images without corresponding reference images, alongside the U45 dataset. This comprehensive testing across diverse datasets ensures that the proposed model’s robustness and adaptability are thoroughly validated in both paired and unpaired scenarios.

#### 4.1.2. Training Details

The model was developed using PyTorch 2.0.0 on a Ubuntu 20.04 of Linux system and trained on an NVIDIA RTX 3090 GPU for 100 epochs with a batch size of 4, ensuring efficient and stable training. Input images were resized to 256 × 256 and standardized with a mean and standard deviation of 0.5 for consistent scaling. Optimization used the AdamW optimizer with default parameters (β_1_ = 0.9, β_2_ = 0.999) and a learning rate decreasing from 1 × 10^−4^ to 1 × 10^−6^ for smooth convergence and reduced overfitting [28].

#### 4.1.3. Evaluation Metric and Benchmark Methods

We employed PSNR (Peak Signal-to-Noise Ratio), SSIM (Structural Similarity Index) [29], UIQM (Underwater Image Quality Measure) [30], and UCIQE (Underwater Color Image Quality Evaluation) [31] to evaluate our method’s performance in enhancing underwater images. PSNR measures distortion, with higher values indicating better noise reduction and detail preservation, while SSIM assesses structural integrity, contrast, and brightness, with values near 1 reflecting improved clarity and fidelity. For non-referenced images, UIQM evaluates colorfulness, sharpness, and contrast, with higher scores signifying superior visual perception, while UCIQE combines color saturation, density, and contrast, with larger values indicating enhanced quality and balanced representation. These metrics comprehensively demonstrate our method’s effectiveness in reducing color distortion and enhancing details.

We compared our method with state-of-the-art approaches, including FUnIEGAN, U-Trans [23], WaterNet [32], UGAN [33], PUGAN [34], and LiteEnhance [35], which utilize advanced techniques like GANs, transformers, and lightweight frameworks. Using their publicly available code and adhering to original settings ensured fair and reproducible evaluations, allowing for a reliable assessment of each method’s strengths and limitations relative to our model.

### 4.2. Quantitative Evaluations

Table 1 provides a detailed quantitative comparison of PCAFA-Net with several state-of-the-art underwater image enhancement methods on the LSUI and UIEB datasets [36]. On the LSUI dataset, PCAFA-Net achieved a PSNR of 27.744 and an SSIM of 0.887, surpassing the performance of all other techniques included in the evaluation. Similarly, on the UIEB dataset, PCAFA-Net demonstrated superior results with a PSNR of 22.801 and an SSIM of 0.890, both of which are the highest values among the compared methods. This demonstrates that our model still holds a significant advantage in preserving image structure.

The quantitative results of the comparison between our proposed PCAFA-Net and several state-of-the-art methods on two non-reference datasets, C60 and U45, are summarized in Table 2. On the C60, PCAFA-Net achieved a UIQM score of 3.01, slightly lower than those of FUnIEGAN and UGAN but still higher than most other methods, indicating a higher image quality. Meanwhile, its UCIQE score is 0.603, the highest among all methods, demonstrating superior color and contrast. On the non-reference dataset U45, its UIQM is 3.269 and its UCIQE is 0.616, both of which are higher than those of other methods.

Moreover, as can be seen from Table 3, the number of parameters in the PCAFA-Net network is at a moderate level, and it has an acceptable processing time. We also included the FLOPs metric of the algorithm, which is slightly higher, but still far lower than WaterNet’s 193.70 G. These also indicate that while PCAFA-Net achieves relatively good performance, it maintains an acceptable level of complexity and computational efficiency.

PCAFA-Net demonstrates exceptional performance across a variety of datasets, consistently achieving top or near-optimal results in key evaluation metrics such as PSNR, SSIM, UIQM, and UCIQE [37]. Furthermore, PCAFA-Net’s strong adaptability to diverse underwater environments underscores its robustness and versatility.

### 4.3. Qualitative Comparsions

**Figure 6:**
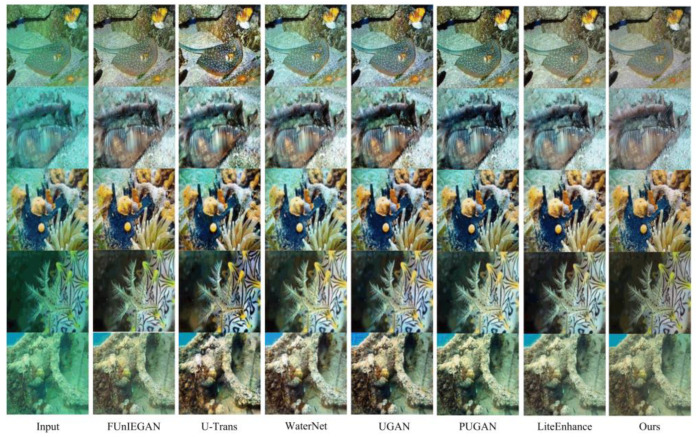
Qualitative comparison results of various methods on the LSUI.

A selection of images from the LSUI test set was randomly picked, and they were tested using the aforementioned methods. These images have varying tones, brightness, and contrast. As shown in the Figure 6, we can observe that FUnIEGAN and U-Trans introduce noticeable noise during the restoration process. While WaterNet, UGAN, and PUGAN perform well in terms of scene contrast, they introduce color distortions. Although LiteEnhance excels in detail restoration, it suffers from some color loss. In the fourth group of comparisons in Figure 6, although PCAFA-Net performs slightly weaker in restoring the extremely low-light region at the top left of the image, the overall restoration result is still quite good. Compared to the results of other methods, our approach demonstrates superior image quality in terms of both image details and color, outperforming other state-of-the-art techniques.

### 4.4. Ablation Analysis

Ablation studies on the LSUI dataset (Table 4) highlight the critical role of AGSM, ACRAM, and FSSAM in our network. Removing any component significantly reduces image quality, causing blurring, structural degradation, and color distortions. The complete model achieves the best performance, excelling in detail preservation, color accuracy, and overall image quality.

## 5. Application

**Figure 7:**
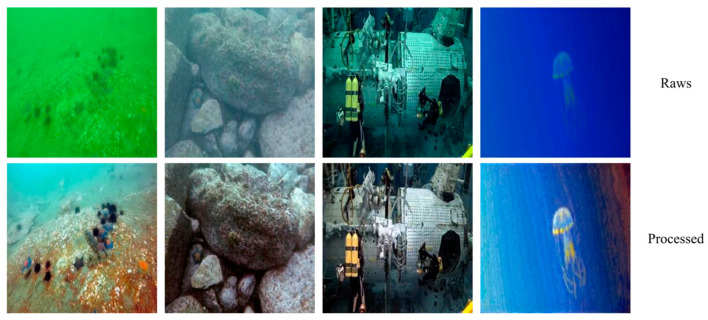
Enhancement on RUOD.

The applications of PCAFA-Net are extensive, with one of the most notable being underwater object detection. When applied to underwater robots, its underwater object detection tasks become much easier. We applied PCAFA-Net to the RUOD underwater object detection dataset. The detection targets in the RUOD dataset include 10 categories such as fish, divers, scallops, squids, jellyfish, and others, facing issues such as light interference, color distortion, and fog effects. The processing results of PCAFA-Net are shown in Figure 7.

Although PCAFA-Net demonstrates excellent overall performance and each module has its own advantages, it inevitably introduces some trade-offs. The AGSM module, based on common physical models, may experience performance degradation when faced with extreme low-light or highly turbid environments, as mentioned in Section 4.3. The ACRAM has strong adaptive adjustment capabilities but may amplify noise in low-contrast regions. The FSSAM, which integrates frequency and spatial attention mechanisms, enhances color correction and detail recovery, but at the cost of some real-time performance, making it less suitable for extremely resource-constrained devices. To address these issues, we plan to further optimize the model in future research to improve its stability and computational efficiency in extreme environments.

## 6. Conclusions

In this paper, we propose an underwater image enhancement model, PCAFA-Net. The network consists of several components, including an Adaptive Gradient Simulation Module (AGSM) that simulates the underwater degradation process. Additionally, we introduce an Adaptive Color Range Adjustment Module (ACRAM), which adaptively adjusts the histogram distribution in the RGB, Lab, and HIS color spaces. Furthermore, we propose a Frequency–Spatial Strip Attention Module (FSSAM), which fully utilizes both frequency-domain and spatial-domain information. Experiments on various underwater image datasets confirm that the proposed PCAFA-Net surpasses current state-of-the-art methods in both quantitative metrics and qualitative assessments. In future work, efforts may be directed towards enhancing the model’s computational efficiency and its ability to adapt to different underwater settings.

## Figures and Tables

**Table 1:** This table presents a quantitative comparison of the proposed method (ours) with existing state-of-the-art methods for underwater image enhancement on the LSUI and UIEB datasets. Bold font represents the highest-performing results.

Method	LSUI		UIEB	
	PSNR	SSIM	PSNR	SSIM
FUnIEGAN	23.054	0.822	19.013	0.793
U-Trans	25.151	0.838	20.747	0.830
WaterNet	24.748	0.861	21.153	0.847
UGAN	24.991	0.850	21.442	0.811
PUGAN	23.895	0.849	21.670	0.818
LiteEnhance	22.502	0.816	22.133	0.883
**Proposed**	**27.744**	**0.887**	**22.801**	**0.890**

**Table 2:** This table presents a quantitative comparison of the proposed method (ours) with existing state-of-the-art methods for underwater image enhancement on the C60 and U45 datasets. Bold font represents the highest-performing results.

Method	C60		U45	
	UIQM	UCIQE	UIQM	UCIQE
FUnIEGAN	3.067	0.586	3.208	0.607
U-Trans	2.861	0.576	3.175	0.571
WaterNet	2.763	0.570	2.899	0.577
UGAN	**3.175**	0.595	3.167	0.609
PUGAN	2.899	0.598	3.189	0.606
LiteEnhance	2.956	0.596	3.093	0.579
**Proposed**	3.008	**0.603**	**3.269**	**0.616**

**Table 3:** This table presents a complexity comparison for difference methods.

Method	Parameters	FLOPs	Time
FUnIEGAN	7.01 M	10.23 G	0.02 s
U-Trans	65.6 M	66.2 G	0.04 s
WaterNet	24.81 M	193.70 G	0.55 s
UGAN	57.17 M	38.97 G	0.06 s
PUGAN	95.66 M	72.05 G	0.23 s
LiteEnhance	0.05 M	2.32 G	0.02 s
Proposed	36.83 M	96.45 G	0.08 s

**Table 4:** Quantitative results of ablation studies.

Setting	PSNR	SSIM
(w/o) AGSM	24.075	0.856
(w/o) ACRAM	25.329	0.872
(w/o) FSSAM	24.670	0.862
Complete proposed method	27.744	0.887

## Data Availability

The data are from public datasets, which are introduced in Section 4.1.1.
